# Expansion With IL-15 Increases Cytotoxicity of Vγ9Vδ2 T Cells and Is Associated With Higher Levels of Cytotoxic Molecules and T-bet

**DOI:** 10.3389/fimmu.2020.01868

**Published:** 2020-08-28

**Authors:** Pia Aehnlich, Ana Micaela Carnaz Simões, Signe Koggersbøl Skadborg, Gitte Holmen Olofsson, Per thor Straten

**Affiliations:** ^1^Department of Oncology, National Center for Cancer Immune Therapy (CCIT-DK), Copenhagen University Hospital Herlev, Herlev, Denmark; ^2^Department of Immunology and Microbiology, Faculty of Health and Medical Sciences, University of Copenhagen, Copenhagen, Denmark

**Keywords:** Vγ9Vδ2 T cells, gamma delta T cells, adoptive cell therapy, IL-15, IL15, cytotoxicity, T-bet, hypoxia

## Abstract

Cancer immunotherapy has shown great advances during recent years, but it has yet to reach its full potential in all cancer types. Adoptive cell therapy (ACT) is now an approved treatment option for certain hematological cancers and has also shown success for some solid cancers. Still, benefit and eligibility do not extend to all patients. ACT with Vγ9Vδ2 T cells is a promising approach to overcome this hurdle. In this study, we aimed to explore the effect of different cytokine conditions on the expansion of Vγ9Vδ2 T cells *in vitro*. We could show that Vγ9Vδ2 T cell expansion is feasible with two different cytokine conditions: (a) 1,000 U/ml interleukin (IL)-2 and (b) 100 U/ml IL-2 + 100 U/ml IL-15. We did not observe differences in expansion rate or Vγ9Vδ2 T cell purity between the conditions; however, IL-2/IL-15-expanded Vγ9Vδ2 T cells displayed enhanced cytotoxicity against tumor cells, also in hypoxia. While this increase in killing capacity was not reflected in natural killer (NK) cell marker or activation marker expression, we demonstrated that IL-2/IL-15-expanded Vγ9Vδ2 T cells were characterized by an increased expression of perforin, granzyme B, and granulysin compared to IL-2-expanded cells. These cytotoxic molecules were not only increased in a resting state, but also released to a greater extent upon target recognition. In contrast, CD107a and cytokine expression did not differ between expansion conditions. However, IL-2/IL-15-expanded Vγ9Vδ2 T cells showed higher levels of transcription factor T-bet expression, which could indicate that T-bet and cytotoxic molecule levels confer the increased cytotoxicity. These results advocate the inclusion of IL-15 into *ex vivo* Vγ9Vδ2 T cell expansion protocols in future clinical studies.

## Introduction

In the combat of disseminated cancer diseases, cancer immunotherapy has proven to be a powerful tool during the last few decades. It is now an approved therapy for a variety of cancers, yielding astonishing long-term responses in some patients (1–4). This illustrates the capability of the immune system to fight cancer and underlines the great potential of immunotherapies. ACT has been particularly successful, in both leukemia and solid cancers, such as malignant melanoma. Still not all patients benefit from this treatment; and over and above, far from all are eligible. Therefore, new approaches are in demand—and in this context, a frequently overlooked cell type is the γδ T cell.

γδ T cells are divided into different subsets based on the usage of the *gamma* and *delta* chains in the T cell receptor (TCR) complex. This study focuses on the Vγ9Vδ2 T cell subset, the most dominant subset in the blood (5), which is restricted to humans, higher primates, and alpacas (6). Target recognition and activation of Vγ9Vδ2 T cells is highly debated in the field. Recognition of phosphoantigens, independent from the major histocompatibility complex (MHC), has long been known to trigger γδ TCR signaling (7). Some phosphoantigens, such as hydroxy-dimethyl-allyl-pyrophosphate (HMBPP), can be exogenously synthesized by bacteria, while other phosphoantigens, such as isopentenyl pyrophosphate (IPP), occur endogenously as by-products of the mevalonate isoprenoid pathway (8). This pathway can be blocked by aminobisphosphonates, like zoledronic acid (ZOL). These market-available drugs inhibit the farnesyl pyrophosphate synthase and thus induce an accumulation of phosphoantigens, among others IPP. This can be exploited for both expansion of Vγ9Vδ2 T cells (9) and augmentation of phosphoantigen presentation on tumor cells (10). Apart from the involvement of butyrophilin proteins, the exact mechanism of the γδ TCR activation by phosphoantigens remains unclear, and conflicting studies have been published in the field (11–13). Since Vγ9Vδ2 T cells express natural killer group 2, member D (NKG2D), and DNAX accessory molecule-1 (DNAM-1) on their surface, they are also activated by NKG2D ligands (14, 15) and DNAM-1 ligands (16). The hierarchy of TCR and NK cell receptor-mediated signaling, however, is controversial (17). Despite the controversies on Vγ9Vδ2 T cell activation, the ability of Vγ9Vδ2 T cells to kill a variety of tumor cells is very evident (18, 19).

For this reason, Vγ9Vδ2 T cells represent an alternative to chimeric antigen receptor (CAR) or TCR-engineered T cells as effector cells in ACT. Like the αβ T cells used for genetic modification, Vγ9Vδ2 T cells can be harvested from blood and easily expanded *ex vivo* with market-approved drugs (9). However, compared to αβ T cells, Vγ9Vδ2 T cells stand out by their broad tumor specificity without the need of engineering: They recognize the altered state of the tumor cell rather than a single tumor antigen—both through NK cell receptors and the γδ TCR. It is well established that the mevalonate pathway is upregulated in many cancers (20), leading to increased phosphoantigen presentation and subsequent Vγ9Vδ2 T cell recognition (10). Finally, since Vγ9Vδ2 T cells perform stress surveillance, they are likely more robust to immune evasion by antigen downregulation and they can also recognize tumors that are not highly mutated, such as acute myeloid leukemia (19). Due to the numerous assets of Vγ9Vδ2 T cells, several clinical trials have been conducted to investigate the safety of ACT with Vγ9Vδ2 T cells as a treatment against solid cancers [as reviewed in (21–23)]. To expand Vγ9Vδ2 T cells, all studies used IL-2 and either synthetic phosphoantigens or ZOL. In most trials, *ex vivo* expanded Vγ9Vδ2 T cells were then re-infused in combination with IL-2 and ZOL or the synthetic phosphoantigen bromohydrin pyrophosphate (BrHPP) (24–26). While all studies considered the treatment as safe and well tolerated, clinical responses were only detected in very few patients. The lack of clinical effectivity could be explained by different factors—e.g., lack of pre-treatment lymphodepletion, difficulties of immune cell homing to the tumor, or an immunosuppressive tumor microenvironment. Alternatively, one of the problems has been suggested to be the functionality of expanded Vγ9Vδ2 T cells *in vivo* (27, 28). Therefore, an infusion product comprising Vγ9Vδ2 T cells of optimal functionality is desirable.

IL-15 is a promising candidate for the improvement of Vγ9Vδ2 T cell expansion. Upon administration of recombinant IL-15 as a monotherapy for patients with metastatic melanoma or renal cell carcinoma, an induction of NK cells and γδ T cells was observed in the blood (29). This strongly points toward a positive effect of IL-15 on cell survival and function in these two cell types. Indeed, the IL-15 signaling pathway has recently been shown to directly contribute to effector functions in human NK cells (30). Despite these encouraging indications, few attempts have been made to test the influence of IL-15 on Vγ9Vδ2 T cell expansion (31, 32).

To achieve an enhanced cell infusion product for ACT with Vγ9Vδ2 T cells, we investigated the effects of different cytokine conditions on Vγ9Vδ2 T cell expansion in this study. Specifically, we compared an expansion condition with high IL-2 to a condition with low IL-2 and additional IL-15. We hypothesize that Vγ9Vδ2 T cell expansion with a combination of IL-2 and IL-15 produces a more cytotoxic infusion product, which could eventually lead to improved clinical responses.

## Materials and Methods

### Peripheral Blood Mononuclear Cell (PBMC) Isolation

Buffy coats from healthy donor blood donations were collected at the blood bank at Rigshospitalet, Copenhagen University Hospital, Denmark. PBMCs were isolated using Lymphoprep^TM^ density gradient media (Alere Technologies) and frozen in fetal bovine serum (FBS, Gibco) with 10% dimethyl sulfoxide (DMSO, Honeywell). Cells were cryopreserved and stored at −150°C in vials of 10–30 × 10^6^ cells.

### Expansion of Vγ9Vδ2 T Cells

Vγ9Vδ2 T cells were expanded from isolated, frozen PBMCs. On day 0 of expansion, 0.5 × 10^6^ PBMCs/ml were seeded in 2 ml of X-Vivo 15 medium (Lonza) with 5% human serum (Sigma Aldrich) per well in a 24-well plate (Corning Costar) and stimulated with 10 μM ZOL (Fresenius Kabi). On day 2 of expansion, cytokine stimulation with either (a) 1,000 U/ml IL-2 (Peprotech) or (b) 100 U/ml IL-2 and 100 U/ml IL-15 (Peprotech) was started. From here, fresh media and cytokines were provided every 2 to 3 days. Purity of Vγ9Vδ2 T cells was examined on days 9, 14, 25, and 50 of culture by flow cytometry analysis. During the 50-day expansion, half of the growing cultures were cryopreserved for later experiments at these time points. Only Vγ9Vδ2 T cell cultures with >90% Vγ9-positive CD3^+^ cells were considered pure and used for further experiments. A total of 12 Vγ9Vδ2 T cell cultures from healthy donors was expanded in this study and named γδHD1-12. Due to timing and cell material availability, not all experiments could be conducted with the same cultures. It is indicated in the figure legends, which cultures were used in the specific experiments.

### Cancer Cell Cultures

The malignant melanoma cell lines FM-28 (ESTDAB-006) and FM-55M1 (ESTDAB-012), the prostate cancer cell line PC-3, and the chronic myelogenous leukemia cell line K562 were cultured in RPMI 1640 GlutaMAX-I^TM^ medium (RPMI, Gibco) supplied with 10% FBS (R10). Prior to intracellular staining or cytotoxicity assays, the cancer cells were left untreated or pre-treated with 10 μM ZOL for 24 h—these are called, e.g., FM-28 or FM-28/ZOL, respectively, in the following.

### Phenotyping of Vγ9Vδ2 T Cells

For flow cytometry analysis, 0.15–1 × 10^6^ cells were placed in flow cytometry tubes and washed with 2 ml of phosphate-buffered saline (PBS) with 2% FBS (FACS buffer). Each sample was stained with 50 μl of antibody mix diluted in FACS buffer for 20–30 min at 4°C in the dark. Afterwards, samples were washed twice, resuspended in FACS buffer, and acquired on the FACS CantoII (BD Biosciences) or the NovoCyte Quanteon (ACEA Biosciences). Analysis was performed with the FACSDiva or NovoExpress software. Generally, lymphocytes were gated first, and then doublets and dead cells were excluded. Vγ9Vδ2 T cells were determined as Vγ9-expressing CD3^+^ cells. For markers without distinct positive and negative population, isotype or fluorescence-minus-one controls were employed to set gates. Antibodies used in this study are listed in Table 1.

**Table 1:** Antibodies used in this study.

**Marker**	**Fluorochrome(s)**	**Clone**	**Company**
**Surface antibodies**			
CD3	AlexaFluor700/BV421	UCHT1	BioLegend
CD4	BV510/BV711	OKT4	BioLegend
CD8	BV605/FITC	SK-1	BioLegend
CD16	PE-Cy7	B73.1	BioLegend
CD19	APC	SJ25C2	BD Biosciences
CD56	PE/PE-Cy7	NCAM15.2	BD Biosciences
CD107a	BV421	H4A3	BD Biosciences
DNAM-1	FITC	11A8	BioLegend
HLA-DR	V500	G46-6	BD Biosciences
NKG2D	PE	1D11	BioLegend
NKp30	APC	AF29-4D12	Miltenyi
NKp44	APC	2.29	Miltenyi
NKp46	APC	REA808	Miltenyi
PD-1	PE-Cy7	EH12.1	BD Biosciences
Vγ9	PE-Cy5	IMMU360	Beckman Coulter
**Intracellular antibodies**			
Granulysin	PE	DH2	BioLegend
Granzyme B	APC	GB-11	ThermoFisher Scientific
IFN-γ	BV510	4S.B3	BioLegend
Perforin	FITC	B-D48	BioLegend
T-bet	PE	4B10	BioLegend
TNF-α	PE-CF594	MAb11	BD Biosciences
**Isotypes**			
**-**	PE-Cy7	MOPC-21	BD Biosciences
**-**	V500	G155-178	BD Biosciences
**Live/dead cell marker**			
LIVE/DEAD Fixable Near IR Dead Cell Stain			ThermoFisher Scientific

### Intracellular Staining

To stain for cytotoxic molecules, degranulation markers, and cytokines upon stimulation, co-cultures with Vγ9Vδ2 T cells and melanoma cells were set up. For this purpose, 0.25 × 10^6^ Vγ9Vδ2 T cells were co-cultured with 0.325 × 10^6^ melanoma cells (FM-28 or FM-28/ZOL) in a 96-well plate (Corning Costar). For 25-day-expanded Vγ9Vδ2 T cells, duplicates of the co-cultures were included, which was not possible for 9- and 14-day-expanded Vγ9Vδ2 T cells due to availability of cell material. Co-cultures were incubated for 5 h in the presence of Brefeldin A (BioLegend) and anti-CD107a antibody. Positive and negative controls were included: Vγ9Vδ2 T cells without stimuli as a baseline/negative control, and Vγ9Vδ2 T cells plus 5 ng/ml PMA (Sigma Aldrich) and 75 nM ionomycin (Sigma Aldrich) as a positive control. For 9- and 14-day-expanded Vγ9Vδ2 T cells, FM-28-co-cultured Vγ9Vδ2 T cells served as the baseline sample. Before surface staining, cells were centrifuged and washed twice with 150 μl of FACS buffer. Duplicate wells of co-cultured cells were pooled to have sufficient amounts of cells to analyze after fixation. Cells were then stained with cell surface antibodies in a volume of 50 μl for 30 min at 4°C in the dark. Subsequently, cells were washed twice, and then fixed and permeabilized as described by the manufacturer (Intracellular Fixation & Permeabilization Buffer Set, eBioscience). In brief, cells were fixated in 200 μl of Fixation Buffer per well overnight at 4°C in the dark. On the next day, cells were washed twice with 150 μl of Permeabilization Buffer and stained with intracellular antibodies as described for surface antibody staining. Lastly, cells were washed twice with Permeabilization Buffer, resuspended in FACS buffer and acquired on the NovoCyte Quanteon. Absolute release of cytotoxic molecules was calculated as the difference of mean fluorescence intensity (MFI) between FM-28-co-cultured and FM-28/ZOL-co-cultured Vγ9Vδ2 T cells [*absolute release* = *MFI*(*FM-28-co-culture*) –* MFI*(*FM-28/ZOL-co-culture*)]. Relative release of cytotoxic molecules was defined as the ratio of the absolute release compared to the MFI of FM-28-co-cultured Vγ9Vδ2 T cells [*relative release* = *absolute release/MFI*(*FM-28-co-culture*)].

For intracellular staining of transcription factor T-bet, 0.3 × 10^6^ (for 9- and 14-day-expanded) or 0.6 × 10^6^ (for 25-day-expanded) Vγ9Vδ2 T cells were plated in a 96-well plate. Washing and surface staining were performed as described in the section above. The fixation/permeabilization procedure was done according to the True-Nuclear^TM^ Transcription Factor Buffer Set (BioLegend). In brief, cells were resuspended in 200 μl of True-Nuclear^TM^ Fix Concentrate per well and incubated for 60 min in the dark at room temperature. Thereafter, three washes with 200 μl of True-Nuclear^TM^ Perm Buffer per well were performed. Anti-T-bet antibody was diluted in True-Nuclear^TM^ Perm Buffer to a total volume of 50 μl per sample. Staining was carried out for 30 min in the dark at room temperature. Before acquiring on the NovoCyte Quanteon, cells were washed three times with True-Nuclear^TM^ Perm Buffer and resuspended in FACS buffer. Data analysis was performed with NovoExpress software.

### xCELLigence Real-Time Cell Analysis Assay

To measure cytotoxicity of Vγ9Vδ2 T cells against cancer cells, the xCELLigence system was used. For this purpose, FM-28, FM-55M1, and PC-3 cells were pre-stimulated with 10 μM ZOL for 24 h and then plated at a concentration of 5,000, 10,000, and 20,000 cells/well, respectively, in 200 μl of R10 in an E-96 plate (ACEA Biosciences). K562 was left untreated and seeded at a density of 20,000 cells/well in an E-96 plate pre-coated with 4 μg/ml anti-CD29 tethering reagent [Leukemic Cell Killing (anti-CD29) Assay, ACEA Biosciences]. The ideal seeding densities were formerly optimized (data not shown). The plate was then inserted into the xCELLigence SP system (ACEA Biosciences) for the cells to attach and initiate proliferation. This period varied between cell lines: 25 h for FM-28 and FM-55M1 or 7 h for K562 and PC-3.Thereafter, the plate was removed from the system and 100 μl of media was removed from each well. Vγ9Vδ2 T cells were added to the plate in 100 μl of R10 at effector-to-target cell ratios 0:1 (as proliferation control) and 1:1. For the FM-28 cell line, effector-to-target cell ratios 1:3 and 3:1 were added, whereas cytotoxicity against K562 was tested at a ratio of 3:1. Additionally, wells with Vγ9Vδ2 T cells alone were included to account for the effector cells' contribution to the cell index. The plate was re-inserted into the system and readout resumed for 72 h. Data were analyzed with the immunotherapy module of the xCELLigence RTCA Software Pro (ACEA Biosciences) as reported previously (33).

To test cytotoxicity under hypoxic conditions, FM-28 cells were cultured in a hypoxic incubator (1% O_2_) for 5 days prior to plating in the xCELLigence DP system. As under normoxic conditions, FM-28 cells were pre-treated with 10 μM ZOL for 24 h and plated at 5,000 cells/well in E-16 plates (ACEA Biosciences). Vγ9Vδ2 T cells were added after 25 h at effector-to-target ratios 0:1 and 1:1 and the experiment was resumed for 72 h after γδ T cell addition.

### Statistical Analyses

Statistical analyses were conducted with the software GraphPad Prism, version 7.00 (GraphPad Software, Inc.). Differences between groups were determined by the Wilcoxon matched-pairs signed-rank test. *P* values smaller than 0.05 were considered significant.

## Results

### Vγ9Vδ2 T Cells Can Be Expanded With High IL-2 as Well as With Low IL-2 in Combination With IL-15

We set out to test *in vitro* expansion of Vγ9Vδ2 T cell cultures under different cytokine conditions, focusing on potential effects on phenotype and functionality. Initially, we aimed to compare stimulation with high levels of IL-2 (1,000 U/ml), low levels of IL-2 (100 U/ml), or combination of IL-2 (100 U/ml) and IL-15 (100 U/ml). However, the Vγ9Vδ2 T cell cultures expanded exclusively with low levels of IL-2 that did not grow and were therefore left out. Thus, only expansion conditions for high level IL-2 and combination of low levels IL-2 and IL-15 were tested.

**Figure 1:**
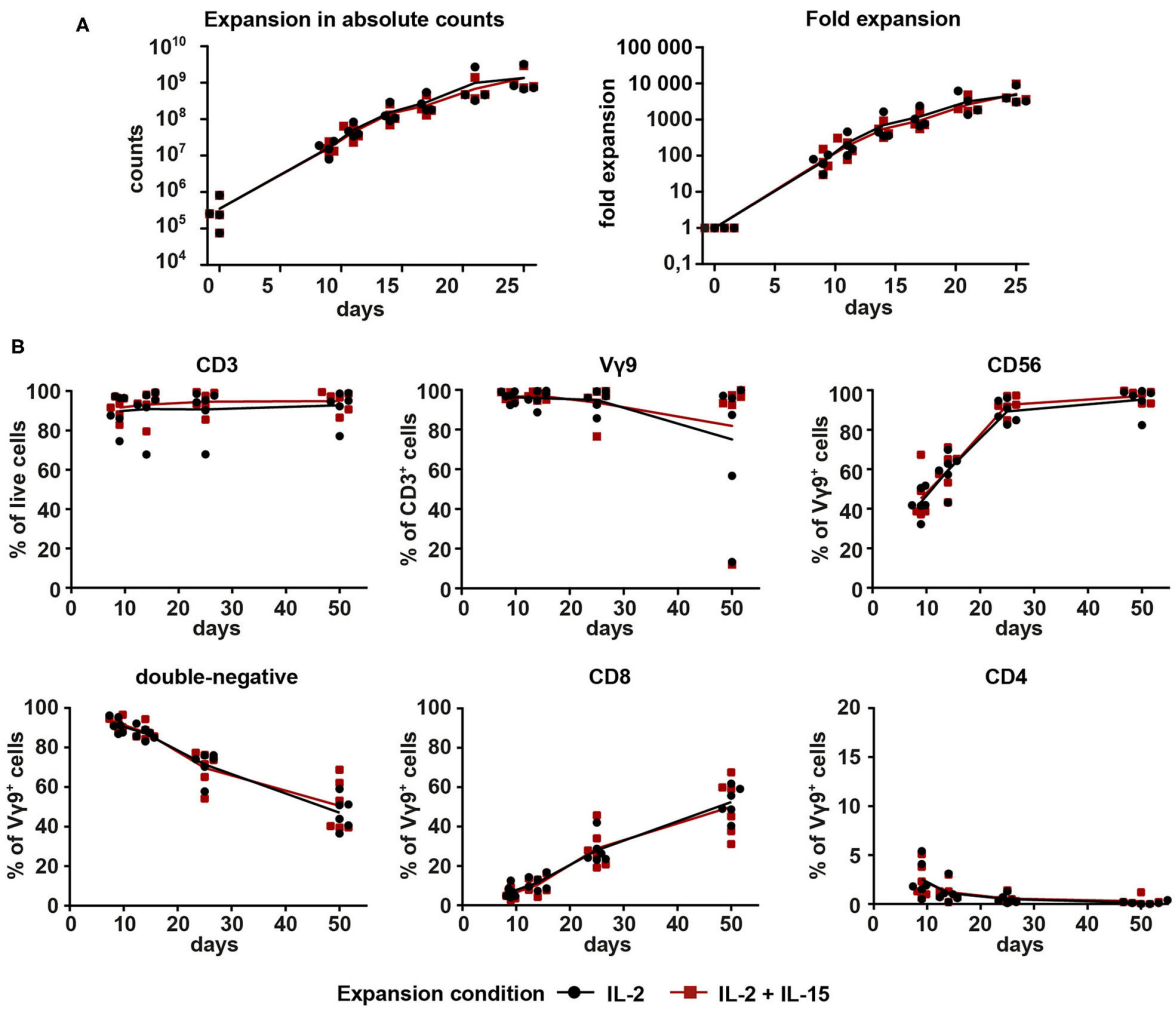
Proliferation and purity analysis of Vγ9Vδ2 T cells expanded with IL-2 (1,000 U/ml) or combined IL-2 (100 U/ml) + IL-15 (100 U/ml). Vγ9Vδ2 T cells were expanded with 10 μM ZOL and either (a) 1000 U/ml IL-2 (black) or (b) 100 U/ml IL-2 + 100 U/ml IL-15 (red). **(A)** Total cell count of Vγ9Vδ2 T cell cultures expanded from PBMCs (*n* = 4, compiled from three independent experiments; γδHD1–4) for 25 days. Fold expansion was calculated based on the number of Vγ9^+^ cells initially present in the culture. **(B)** Vγ9Vδ2 T cell cultures expanded from PBMCs (*n* = 6, compiled from three independent experiments; γδHD5–10) for 50 days were analyzed by flow cytometry for expression of CD3, Vγ9, CD56, CD4, and CD8 on days 9, 14, 25, and 50 of culture. Lymphocytes were gated first, and then doublets were excluded before gating on live cells. Lines represent means of all cultures.

To compare the two expansion conditions for Vγ9Vδ2 T cell cultures, PBMCs from four different healthy donors were initially stimulated with 10 μM ZOL on day 0. From day 2 onward, cultures were given either 1,000 U/ml IL-2 (IL-2-expanded cells) or 100 U/ml IL-2 + 100 U/ml IL-15 (IL-2/IL-15-expanded cells) for a total of 25 days (Figure 1A). Having started out with a median of 2.5 × 10^5^ Vγ9^+^ cells (range 0.75–8.2 × 10^5^) on day 0, cell expansions reached a median total number of 810 × 10^6^ Vγ9^+^ cells (range 672–3230 × 10^6^) on day 25. This corresponded to a median fold expansion of 3765-fold (range 3051- to 9718-fold), showing no difference between the two expansion conditions. Purity of Vγ9Vδ2 T cells was controlled by flow cytometry on days 0, 9, 14, and 25. Vγ9Vδ2 T cell cultures were considered pure at >90% of CD3^+^ cells showing Vγ9 surface expression.

After this first successful expansion of Vγ9Vδ2 T cells, we decided to extend the expansion period to 50 days. Purity of the cultures was monitored on days 9, 14, 25, and 50 to be able to compare the two expansion conditions. Overall, no differences between the two conditions were detected (Figure 1B). CD3 expression remained stably high for the course of 50 days for all six healthy donors. Three expansion cultures (two IL-2-expanded, one IL-2/IL-15-expanded) dropped in expression of the Vγ9 chain between days 25 and 50, but the remaining cultures were highly pure throughout. The three outlier cultures with low Vγ9 expression were dominated by CD3^+^ cells with a CD8 expression of around 95% (data not shown). The cytotoxicity and NK lineage marker CD56 increased drastically on Vγ9^+^ cells in the first 25 days and then stabilized to a median of 98.4% CD56 expression. Regarding the expression of co-receptors CD4 and CD8, the majority of Vγ9^+^ cells (>85%) was double-negative for both markers during the early stages of the culture. This proportion, however, decreased over time in favor of an increasing amount of CD8^+^ cells. A small fraction of CD4^+^Vγ9^+^ cells (between 0.5 and 5.4%) could be detected on day 9, with a tendency to decrease with time. Overall, data in Figure 1 shows that expansion of Vγ9Vδ2 T cells is feasible with both expansion conditions, 1,000 U/ml IL-2 as well as 100 U/ml IL-2 + 100 U/ml IL-15. In addition, no differences between conditions could be detected in terms of expansion rates or purity.

### IL-2/IL-15-Expanded Vγ9Vδ2 T Cells Kill Cancer Cells Faster and More Efficiently

**Figure 2:**
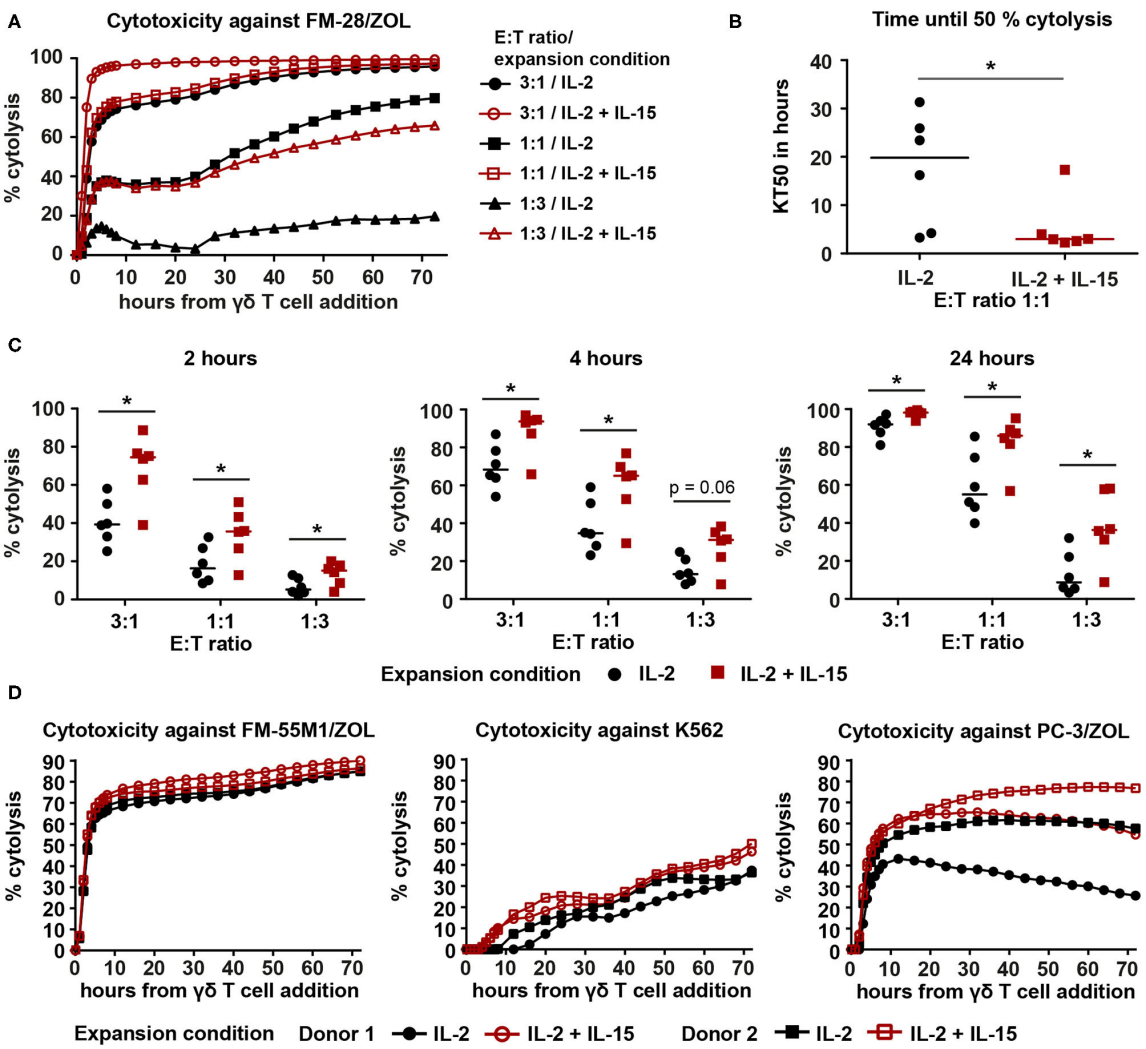
IL-2/IL-15-expanded Vγ9Vδ2 T cells show enhanced cytotoxicity against various cancer cell lines in a 72-h xCELLigence real-time cell analysis assay. **(A)** Representative cytolysis plot, comparing Vγ9Vδ2 T cell ability to kill FM-28/ZOL between the two expansion conditions. The experiment was conducted at three different effector–target cell ratios (3:1, 1:1, 1:3). Black symbols belong to IL-2-expanded cultures, whereas red symbols indicate IL-2/IL-15-expanded cultures. Lines represent means of three technical replicates. **(B)** Time to kill 50% of the target tumor cells (KT50) was compared between both expansion conditions at an effector–target cell ratio of 1:1. Horizontal lines depict the median KT50 of the respective expansion conditions (*n* = 6, compiled from two independent experiments, γδHD7–12). **(C)** Comparison of percentage of cytolysis at time points 2, 4, and 24 h after addition of IL-2-expanded vs. IL-2/IL-15-expanded Vγ9Vδ2 T cells. Percentage of cytolysis is shown for different effector–target cell ratios. Horizontal lines indicate median cytolysis (*n* = 6, compiled from two independent experiments, γδHD7–12). **(D)** Cytolysis plots comparing Vγ9Vδ2 T cell ability to kill FM-55M1/ZOL (left plot), K562 (middle plot), and PC-3/ZOL (right plot) between expansion conditions. Cytotoxicity was assessed at an effector–target cell ratio of 1:1 for FM-55M1/ZOL and PC-3/ZOL and at a ratio of 3:1 for K562. Black, filled symbols indicate IL-2-expanded cultures, whereas matching red, empty symbols indicate the associated IL-2/IL-15-expanded cultures (*n* = 3 with *n* = 2 per cell line, compiled from two independent experiments; γδHD9,10,12). % cytolysis and KT50 were calculated using the xCELLigence RTCA Software Pro (ACEA Biosciences). Statistical significance was determined with the Wilcoxon matched-pairs signed-rank test. *p < 0.05. FM-28, FM-55M1 = melanoma cell lines. K562 = chronic myelogenous leukemia cell line. PC-3 = prostate cancer cell line. /ZOL = 24-h-pre-stimulation with ZOL.

It was recently shown that addition of IL-15 to the expansion of Vγ9Vδ2 T cells leads to enhanced cytotoxicity (32). Therefore, we investigated if our IL-2/IL-15-expanded Vγ9Vδ2 T cells were also capable of improved cancer cell killing. For this purpose, Vγ9Vδ2 T cells, cryopreserved on day 25 of culture, were thawed and set up in an xCELLigence real-time cell analysis assay. Melanoma cell line FM-28, which had been pre-treated with 10 μM ZOL to increase phosphoantigen expression, was used as target cell, since non-stimulated FM-28 cells were not killed by Vγ9Vδ2 T cells (data not shown). As depicted in Figure 2A, cytolysis of FM-28/ZOL cells was rapid during the first 5–6 h and then slowed down. In all six cultures tested, IL-2/IL-15-expanded cells were found to kill 50% of the target cells significantly faster when compared to IL-2-expanded cells (Figure 2B, for effector–target cell ratio 1:1). When comparing cytotoxicity at different time points throughout the assay, we observed that IL-2/IL-15-expanded cells showed increased killing of target cells for all ratios at 2, 4, and 24 h (Figure 2C). This difference in FM-28/ZOL-directed cytotoxicity between IL-2-expanded and IL-2/IL-15-expanded cells was also detectable for 9- and 14-day-expanded Vγ9Vδ2 T cells, albeit less evidently, especially for day 9 (Supplementary Figure 1A). Furthermore, we could show that the enhanced cytotoxicity of IL-2/IL-15-expanded cells extended to other cancer cell lines, also of different origin (Figure 2D). While dynamics of killing of melanoma cell line FM-55M1/ZOL and prostate cancer cell line PC-3/ZOL resembled those of FM-28, cytolysis was more gradual for chronic myelogenous leukemia cell line K562, which was not pre-treated with ZOL to mimic a more NK cell-like response. Also, in this less TCR-dependent manner of tumor recognition, IL-2/IL-15-expanded cells performed better than IL-2-expanded cells. This clearly demonstrated a superiority of IL-2/IL-15-expanded cells over IL-2-expanded cells in terms of cytotoxicity against a variety of cancer cells.

### No Differences in Phenotype Between IL-2-Expanded and IL-2/IL-15-Expanded Vγ9Vδ2 T Cells

**Figure 3:**
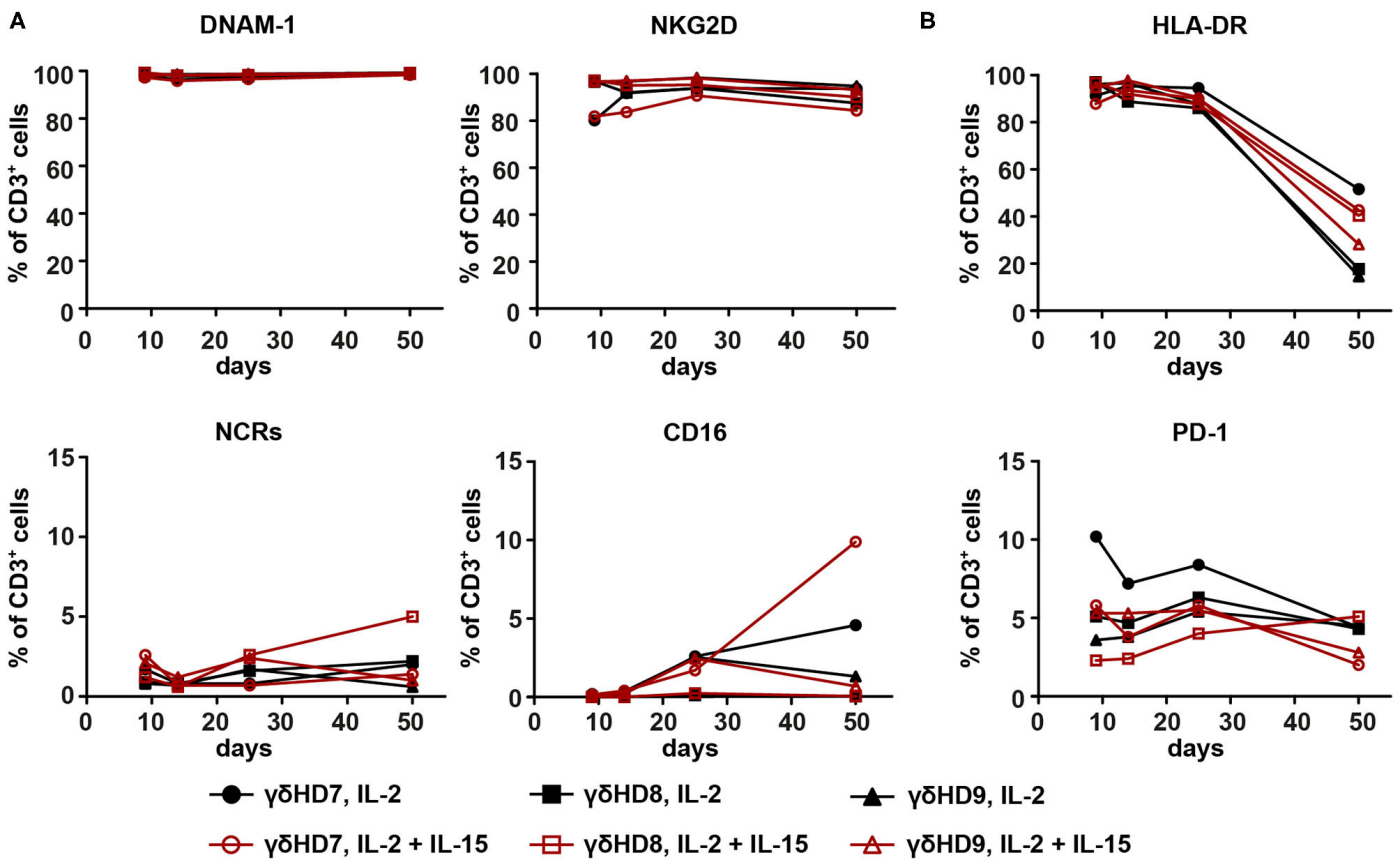
Phenotype analysis of NK cell and activation markers of expanded Vγ9Vδ2 T cell cultures did not reveal differences between IL-2-expanded and IL-2/IL-15-expanded cultures. The three purest Vγ9Vδ2 T cell cultures from the 50-day expansions were analyzed by flow cytometry for **(A)** NK cell markers DNAM-1, NKG2D, NCRs (NKp30, NKp44, NKp46), and CD16, as well as **(B)** activation markers HLA-DR and PD-1. CD3^+^ cells were gated from single, live lymphocytes. Due to the high purity of cultures (>95% Vγ9^+^ of CD3^+^ cells), no γδ T cell marker was included. HLA-DR- and PD-1-positive cells were gated with the help of isotype controls; NCR-positive cells with a fluorescence-minus-one control (*n* = 3 for both expansion conditions, representative data from two independent experiments; γδHD7–9).

To investigate if discrepancies in phenotype could be associated with the observed differences in cytotoxicity, the three purest Vγ9Vδ2 T cell cultures were subjected to phenotype analysis by flow cytometry. As NK cell receptors have been implicated to play a role in triggering cytotoxicity by Vγ9Vδ2 T cells (15, 16), surface expression of DNAM-1, NKG2D, the natural cytotoxicity receptors (NCRs, measured as a pool of NKp30, NKp44, and NKp46), and CD16 was measured (Figure 3A). Both DNAM-1 and NKG2D were stably expressed at high levels throughout the entire duration of the culture: DNAM-1 expression was >95%, while >80% of cells expressed NKG2D. The NCRs also showed stable expression, although at much lower levels (<5%). While CD16 expression could not be detected in some cultures, others did express small amounts of CD16 varying from 0 to 10%. None of these markers exhibited apparent differences between IL-2-expanded and IL-2/IL-15-expanded cultures. Similarly, no differences between expansion conditions were detected for the tested activation markers (Figure 3B). HLA-DR surface expression was very high during the first 25 days of culture and dropped toward day 50, going from above 87% of cells expressing HLA-DR to 14–52%. In contrast, Vγ9Vδ2 T cells expressed only low levels of PD-1 during the whole culturing time, ranging from 2 to 11%.

While phenotyping could not explain differences in cytotoxicity between expansion conditions, it did contribute with knowledge on the long-term expression of different NK cell and activation markers on expanded Vγ9Vδ2 T cells.

### Higher Levels of Cytotoxic Molecules in IL-2/IL-15-Expanded Vγ9Vδ2 T Cells

We set up an intracellular staining assay to investigate if the production or release of cytotoxic molecules was affected by expansion with IL-15. For this purpose, we cultured Vγ9Vδ2 T cells for 5 h in the presence of Brefeldin A and anti-CD107a antibody in different conditions: (i) with cell culture medium (as a negative/baseline control), (ii) with FM-28 melanoma cells, (iii) with FM-28/ZOL melanoma cells, or (iv) with PMA/ionomycin (as a positive control).

**Figure 4:**
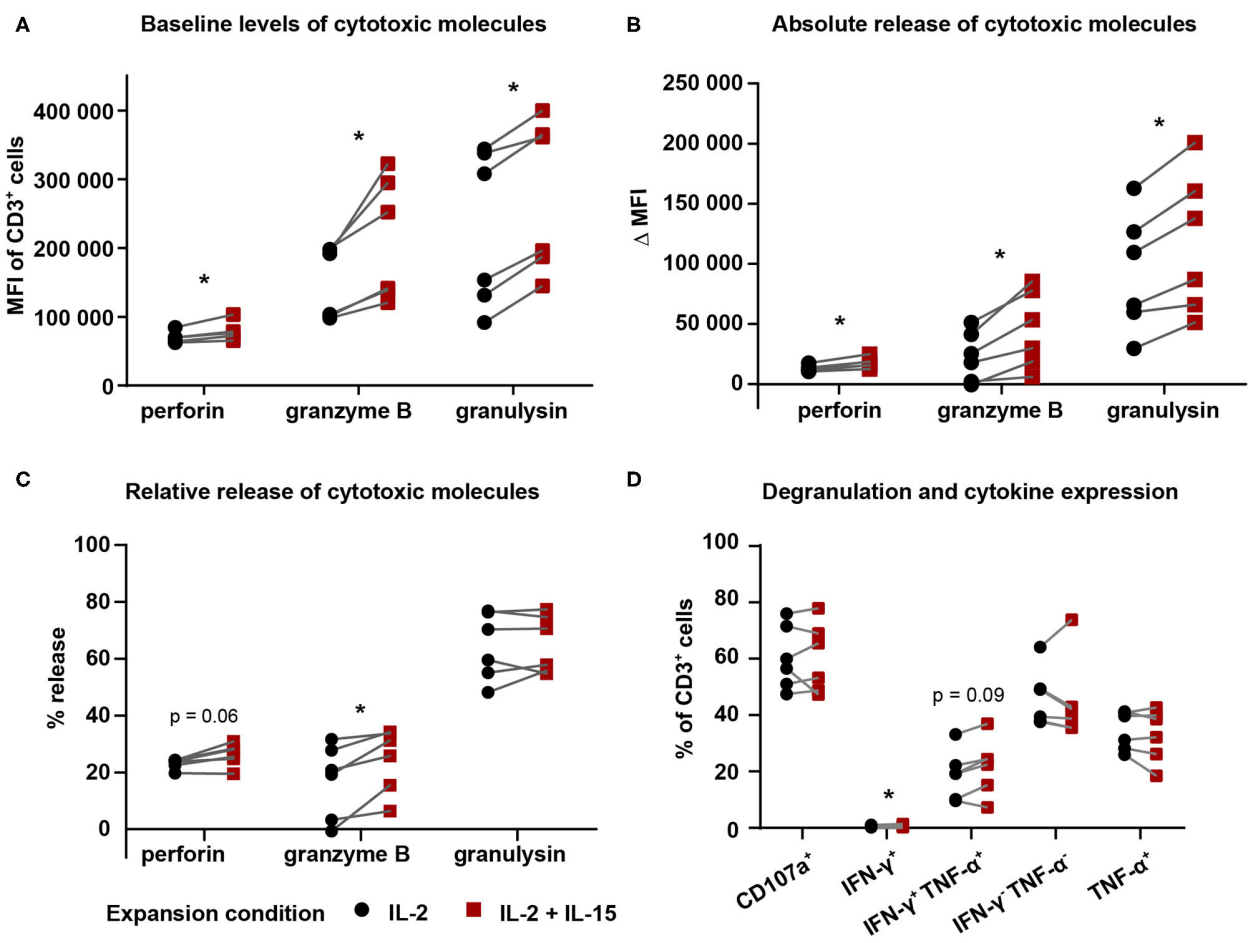
IL-2/IL-15-expanded Vγ9Vδ2 T cells displayed higher levels and release of cytotoxic molecules. Vγ9Vδ2 T cells cryopreserved on day 25 were thawed and co-cultured in the presence of Brefeldin A and anti-CD107a antibody for 5 h in the following conditions: (i) cell culture medium (baseline/negative), (ii) FM-28, or (iii) FM-28/ZOL. **(A)** Intracellular flow analysis was used to determine baseline levels of perforin, granzyme B, and granulysin in Vγ9Vδ2 T cell cultures comparing both expansion conditions. **(B)** Absolute release of cytotoxic molecules (perforin, granzyme B, and granulysin) by Vγ9Vδ2 T cells comparing IL-2-expanded and IL-2/IL-15-expanded cells. Absolute release was calculated based on the difference in MFI between FM-28-co-cultured and FM-28/ZOL-co-cultured Vγ9Vδ2 T cells. **(C)** Relative release of cytotoxic molecules was calculated as the ratio of absolute release compared to the MFI of FM-28-co-cultured Vγ9Vδ2 T cells. **(D)** Degranulation and cytokine expression of IL-2-expanded vs. IL-2/IL-15-expanded Vγ9Vδ2 T cells was determined by gating on positive cells in FM-28/ZOL-co-cultured Vγ9Vδ2 T cells. Gates were set according to the FM-28-co-cultured control. Since all cultures contained >90% Vγ9^+^ cells of CD3^+^ cells, no γδ T cell marker was included in this panel. Instead, CD3 positivity was used to mark Vγ9Vδ2 T cells. The Wilcoxon matched-pairs signed-rank test was applied to determine statistical significant differences between groups. Non-significant *p* values are not shown. *p < 0.05, MFI = mean fluorescence intensity (*n* = 6 in one experiment; γδHD7–12).

Treating cells with only cell culture medium revealed that baseline levels of cytotoxic molecules perforin, granzyme B, and granulysin were different between expansion conditions for Vγ9Vδ2 T cells expanded for 25 days (Figure 4A): IL-2/IL-15-expanded Vγ9Vδ2 T cells displayed higher levels of perforin, granzyme B, and granulysin than IL-2-expanded cells. To investigate the dynamics of cytotoxic molecule release, we calculated the absolute and relative release of perforin, granzyme B, and granulysin. Here, we defined absolute release as the difference in MFI between FM-28-co-cultured Vγ9Vδ2 T cells and FM-28/ZOL-co-cultured Vγ9Vδ2 T cells—representing the absolute difference in molecule number without target recognition (FM-28 co-culture) and with target recognition (FM-28/ZOL co-culture). It could thus be shown that the absolute release of cytotoxic molecules upon target killing was greater in the IL-2/IL-15-expanded Vγ9Vδ2 T cells than in IL-2-expanded Vγ9Vδ2 T cells (Figure 4B). Relative release was determined as the ratio of the absolute release compared to the MFI of the FM-28-co-cultured Vγ9Vδ2 T cells. As seen in Figure 4C, the relative release of cytotoxic molecules was only significantly higher for granzyme B in IL-2/IL-15-expanded cells. A similar trend was observed for perforin, whereas no clear tendency was noted for granulysin. These observations were underlined by the lack of difference in expression of degranulation marker CD107a between IL-2- and IL-2/IL-15-expanded cells (Figure 4D). Thus, it seemed as if cells from both expansion conditions degranulated to a similar degree and released a similar proportion of cytotoxic molecules, but that the granules of IL-2/IL-15-expanded cells contained more cytotoxic molecules at baseline. The differences in baseline levels and absolute release of perforin and granzyme B were preserved in Vγ9Vδ2 T cells expanded for 14 days but seemed to fade out when only expanded for 9 days (Supplementary Figures 1B,C). Relative release of perforin and granzyme B in 9- and 14-day-expanded Vγ9Vδ2 T cells was also similar to 25-day-expanded cells (Supplementary Figure 1D). In contrast, granulysin levels and release did not differ between expansion conditions neither at day 9 nor at day 14. In line with results from day 25, no differences in CD107a expression were found in Vγ9Vδ2 T cells cryopreserved at earlier stages (Supplementary Figure 1E).

Regarding cytokines, we did not detect a biologically relevant difference in expression of interferon (IFN)-γ or tumor necrosis factor (TNF)-α in 25-day-expanded Vγ9Vδ2 T cells between the two expansion conditions, which indicates that these cytokines did not play a crucial role in mediating increased cytotoxicity. The same was valid for 9-day-expanded γδ T cells, whereas a slight increase in IFN-γ-TNF-α-double-positive cells could be noted for IL-2/IL-15-expanded γδ T cells at day 14 (Supplementary Figure 1E).

### IL-2/IL-15-Expanded Vγ9Vδ2 T Cells Are Associated With an Increased T-bet Expression

**Figure 5:**
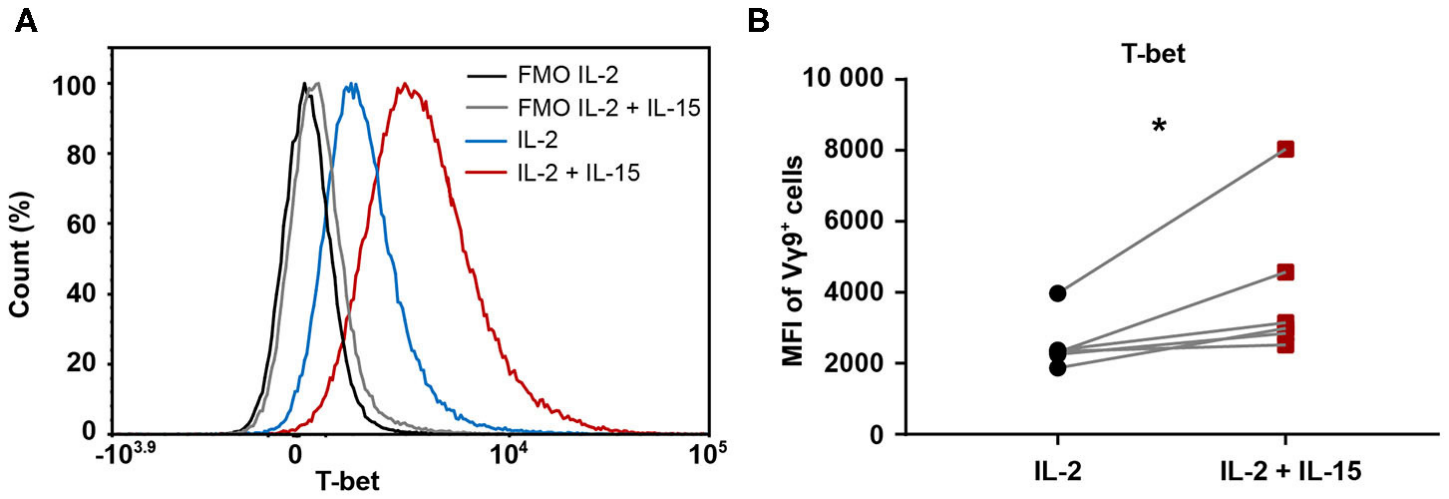
Expression of transcription factor T-bet is increased when including IL-15 during expansion of Vγ9Vδ2 T cells. 25-day-expanded Vγ9Vδ2 T cells (*n* = 6, compiled data from two independent experiments; γδHD1–4, 11–12) were tested for level of T-bet expression by intracellular staining. **(A)** Representative histogram from one healthy donor displaying T-bet expression. Fluorescence-minus-one (FMO) controls are marked in black (IL-2) and gray (IL-2/IL-15), while fully stained samples for IL-2-expanded cells are depicted in blue and IL-2/IL-15-expanded cells in red. **(B)** T-bet expression in IL-2 vs. IL-2/IL-15-expanded cultures, determined by MFI of Vγ9^+^ cells. Significance of differences between groups were calculated with the Wilcoxon matched-pairs signed-rank test. *p < 0.05, MFI = median fluorescence intensity.

A connection between increased amounts of cytotoxic molecules and an enhanced cytotoxicity has previously been suggested in association with a greater expression of the transcription factor T-bet (34). Therefore, we investigated differences in T-bet level between the two expansion conditions. To this end, 25-day-expanded Vγ9Vδ2 T cells cultures were stained for T-bet intracellularly. This revealed a clear right shift in T-bet expression in IL-2/IL-15-expanded cells compared to IL-2-expanded cells, as depicted for a representative donor in Figure 5A. This phenomenon held true for all six healthy donors investigated (Figure 5B), indicating that IL-2/IL-15-expanded Vγ9Vδ2 T cells express higher levels of T-bet than IL-2-expanded cells. While the increased T-bet expression in IL-2/IL-15-expanded cells was not yet visible in 9-day-expanded Vγ9Vδ2 T cells, it was very evident in 14-day-expanded cells (Supplementary Figure 1F).

### Enhanced Cytotoxicity of IL-2/IL-15-Expanded Vγ9Vδ2 T Cells Is Retained Under Hypoxic Conditions

**Figure 6:**
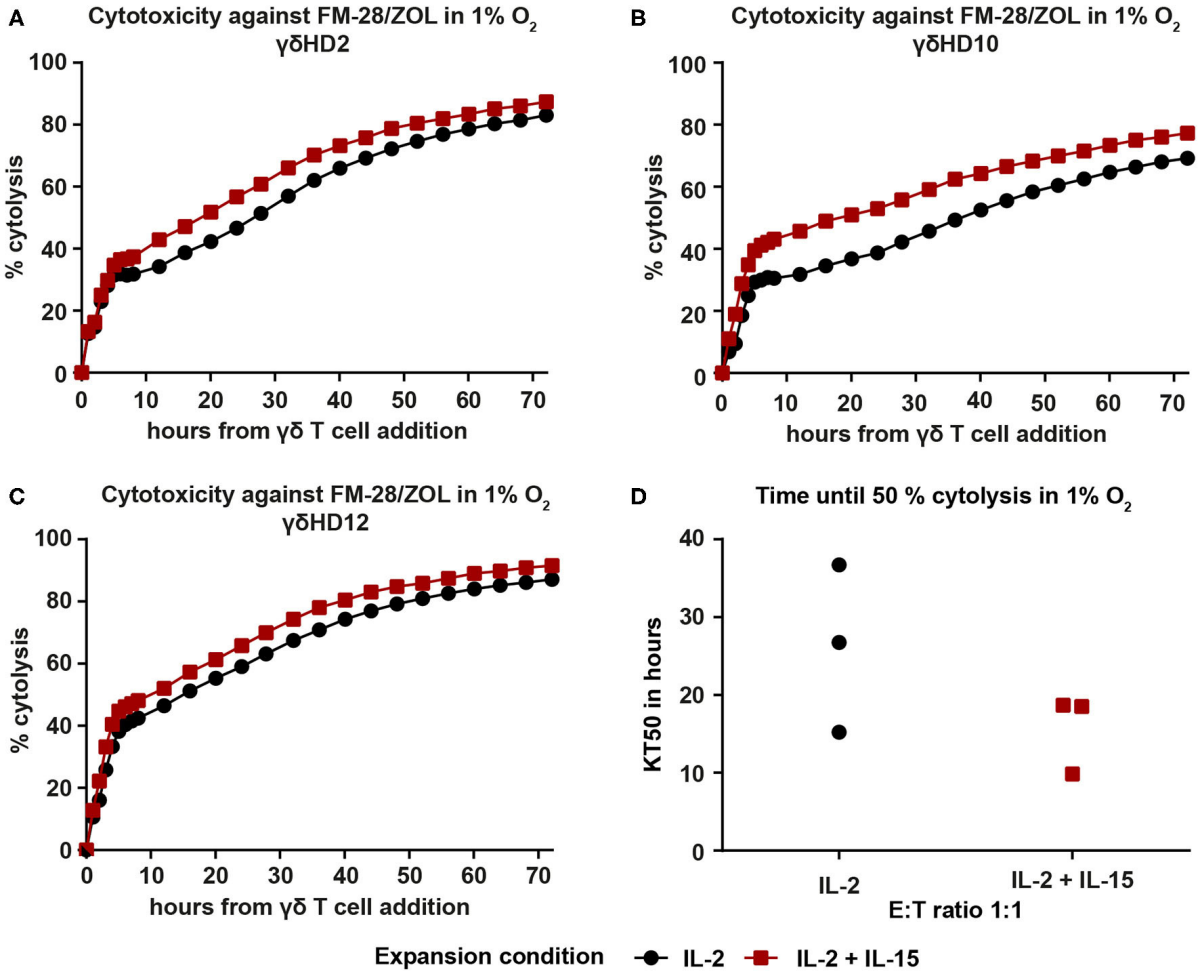
IL-2/IL-15-expanded Vγ9Vδ2 T cells retain increased cytotoxicity against FM-28/ZOL under hypoxic conditions. FM-28 cells were cultured in hypoxia for 5 days prior to seeding in the xCELLigence and stimulated with 10 μM ZOL for the last 24 h. 25-day-expanded Vγ9Vδ2 T cells were added at an effector–target ratio of 1:1 and cytolysis monitored for 72 h in 1% O_2_. Cytolysis plots of donors **(A)** γδHD2, **(B)** γδHD10, and **(C)** γδHD12 comparing cytotoxicity against FM-28/ZOL between expansion conditions in 1% O_2_. Black, round symbols mark IL-2-expanded cultures, while red, square symbols mark IL-2/IL-15-expanded cultures. **(D)** Time to kill 50% of target cells FM-28/ZOL (KT50) is depicted for IL-2-expanded and IL-2/IL-15-expanded Vγ9Vδ2 T cell at an effector–target ratio 1:1 (*n* = 3 in one experiment; γδHD2,10,12). % cytolysis and KT50 were calculated with the xCELLigence RTCA Software Pro.

To examine whether the observed superiority of IL-2/IL-15-expanded Vγ9Vδ2 T cells persists in conditions more resembling those of the tumor microenvironment, we repeated the cytotoxicity assays against ZOL-pre-treated FM-28 cells in hypoxia (1% O_2_). FM-28 cells were cultured in hypoxia for 5 days prior to plating in the xCELLigence system to allow for adaptations to a hypoxic state. For all three donors tested in hypoxia, IL-2/IL-15-expanded Vγ9Vδ2 T cells showed higher cytotoxicity against FM-28/ZOL than IL-2-expanded cells (Figures 6A–C). This is also well illustrated by the time until 50% cytolysis of target cells, since IL-2-expanded cells needed 15–36 h to kill 50% of FM-28/ZOL cells, as opposed to 9–18 h for IL-2/IL-15-expanded cells (Figure 6D). Thus, it appears that the advantage of IL-2/IL-15-expanded Vγ9Vδ2 T cells in cytotoxicity is also preserved under low oxygen conditions.

## Discussion

While cancer immunotherapy has shown great clinical advances over the last years, its potential is still not fully exploited. ACT using T cells equipped with CARs targeting CD19 has been approved for the treatment of selected B cell malignancies. It is highly likely that ongoing clinical trials in onco-hematology will show survival benefit, and thus expand on the numbers of malignant conditions that can be treated with ACT in the future. However, in solid cancer, a key problem is related to the specific targeting of cancer cells, and the related risk of serious side effects. Cellular therapy with Vγ9Vδ2 T cells could be the solution to these problems: They recognize tumors in an MHC-independent, but tumor-specific manner and are easy to harvest from blood. To date, clinical studies with Vγ9Vδ2 T cells have, however, struggled to show compelling clinical responses, which highlights the need for further research to fully harness the capacity of Vγ9Vδ2 T cells as a highly effective cancer killers in immunotherapy.

To improve outcome of adoptive Vγ9Vδ2 T cell therapy, IL-15 has been tested during cell expansion in different setups (31, 32, 35–37). In this study, we investigated the effect of combined IL-2 and IL-15 at low concentrations on the expansion of Vγ9Vδ2 T cells. While a combination of low IL-2 and IL-15 was previously explored, it was only compared to expansion with low IL-2 alone (32). Low IL-2 alone did not allow for Vγ9Vδ2 T cell expansion in our hands; thus, we compared to expansion with high IL-2. The higher concentration of IL-2 could explain why we did not see an increased proliferation of IL-2/IL-15-expanded Vγ9Vδ2 T cells compared to IL-2-expanded cells, which was shown by van Acker and colleagues (32). A novelty of our work is the successful long-term expansion of Vγ9Vδ2 T cells over 50 days, only providing cytokines after the initial stimulation with ZOL. We yet decided to focus our study on 25-day-expanded Vγ9Vδ2 T cells, as we deemed this expansion timeframe most realistic in a clinical setting.

In line with previous findings (32), we observed superior cytotoxicity in IL-2/IL-15-expanded Vγ9Vδ2 T cells over IL-2-expanded cells. However, the previously reported results were based on 4-hour cytotoxicity assays. By measuring cytotoxicity in a 72-hour xCELLigence real-time assay, we have shown that increased killing seems to be maintained beyond the period observed in a short-term cytotoxicity assay. This reflects the clinical situation more accurately and could, thus, also imply a sustained effectivity of Vγ9Vδ2 T cells upon administration to patients.

Next, we were curious to find out what underlies the stronger cytotoxicity in IL-2/IL-15-expanded Vγ9Vδ2 T cells; therefore, we conducted phenotyping and intracellular staining assays. Our investigations into NK cell and activation marker phenotypes did not reveal any differences between expansion conditions, hence not providing an explanation for distinctive levels of cytotoxicity. The lack of impact of IL-15 on the expression of NK cell receptors was unexpected, since IL-15 is implicated in NK cell differentiation (38, 39). Regarding activation markers, we observed low and stable PD-1 expression in both our expansion conditions. Observed PD-1 levels on γδ T cells seem to vary across studies (40–42), and our expression levels join the lower ranks of the spectrum. Studies in humanized NOD scid gamma mice indicate that due to the low PD-1 expression on Vγ9Vδ2 T cells, PD-1 is not of much concern to the effectivity of adoptive Vγ9Vδ2 T cell therapy (43). Still, this remains to be verified in a human *in vivo* setting. Like phenotyping, intracellular staining of cytokines upon co-culture with cancer cells did not prompt any explanations about the enhanced cytotoxicity in IL-2/IL-15-expanded cells. We did not observe any biologically relevant differences in IFN-γ or TNF-α expression between expansion conditions, which indicates that these cytokines do not play a decisive role in mediating the difference in cytotoxicity. On the contrary, others have observed higher IFN-γ levels in IL-2/IL-15-expanded Vγ9Vδ2 T cells compared to IL-2-expanded cells upon co-culture with Daudi or U266 cells (32). However, as the reported cytokine concentrations were very low, biological relevance of the observed differences is uncertain.

Apart from cytokines, we also investigated levels of degranulation and release of cytotoxic molecules by staining for CD107a expression as well as perforin, granzyme B, and granulysin in our intracellular staining assays. While we did not detect any differences in CD107a expression upon target cell killing, we could observe an upregulation of cytotoxic molecules in IL-2/IL-15-expanded Vγ9Vδ2 T cells. Perforin was previously shown to be upregulated in Vγ9Vδ2 T cells upon IL-15 administration (31). The upregulation of cytotoxic molecules was present at baseline, but an increased absolute release was also registered upon co-culture with cancer cells. From this, we inferred that IL-2-expanded and IL-2/IL-15-expanded cells do not differ in their extent of degranulation, but in their quantitative granule content. In other words, it appears that lytic granules of IL-2/IL-15-expanded cells contain more cytotoxic molecules, which render the cells more efficient in killing potential target cells. Interestingly, we could detect the release of granulysin upon cancer cell recognition—this has previously only been associated with γδ T cell-mediated disease control of malaria and tuberculosis infections (44, 45). In general, we hypothesize that the increased amounts of cytotoxic molecules are responsible for the improved cytotoxicity in IL-2/IL-15-expanded cells. This hypothesis is supported by several lines of evidence: Firstly, Wolint et al. studied a mouse model, in which they could distinguish different effector and memory T cell subsets by their cytolytic capacities (46). In their study, extent of degranulation, measured by CD107a expression, did not represent degree of cytotoxicity. In contrast, granule content, determined by granzyme B content, did correlate with cytotoxic capacity, which falls in line with our hypothesis. Secondly, a potential connection between increased levels of cytotoxic molecules in T cells and an enhanced ability to kill has previously been demonstrated in the context of human immunodeficiency virus (HIV) infections (47). This connection was specifically detected in elite controllers, a subset of HIV patients, in whom the viral load is spontaneously controlled. The virus control in these individuals is presumably exerted by the granule-exocytosis pathway of CD8^+^ T cells, as these cells were shown to kill more autologous HIV-infected CD4^+^ T cells than those of progressing patients (47). Finally, Hersperger et al. showed that HIV-specific effector CD8^+^ T cells in elite controllers expressed higher levels of perforin and granzyme B. This was also positively correlated with expression of transcription factor T-bet (34), which led us to investigate the T-bet level in Vγ9Vδ2 T cells as well.

Intracellular staining of T-bet revealed that IL-2/IL-15-expanded Vγ9Vδ2 T cells harbored higher levels of T-bet than IL-2-expanded cells. This could indicate that T-bet plays a role in triggering cytotoxicity programs in the cells, potentially downstream of IL-15 signaling. This notion is supported by Wang et al. (30), who demonstrated a signaling pathway linking IL-15 and T-bet in human NK cells. They showed that IL-15 bound to IL-15Rα triggered the phosphorylation of Akt, which in turn led to the de-ubiquitination of transcription factor functionally spliced X-box binding protein 1 (XBP1s). This transcription factor was then shown to accumulate in the nucleus and to recruit T-bet to the granzyme B promoter, where XBP1s has a direct binding site, but T-bet has not. This resulted in increased amounts of granzyme B protein, which in their experiments also conferred an enhanced cytolytic activity toward leukemia cell lines. Since we also observed higher amounts of granzyme B protein, increased T-bet expression and stronger cytotoxicity upon culturing cells with IL-15, it is conceivable that a similar mechanism is present in Vγ9Vδ2 T cells.

While we observed higher T-bet levels in IL-2/IL-15-expanded Vγ9Vδ2 T cells than in IL-2-expanded cells in a resting state, expression of T-bet could be subject to change in other conditions, e.g., hypoxia. Low oxygen levels were previously shown to dampen T-bet via hypoxia-inducible factor 1α upregulation (48); in general, hypoxia was found to enhance immunosuppressive pathways in the tumor and to interfere with T cell function (49). In light of this, we investigated if the cytotoxic advantage of IL-2/IL-15-expanded Vγ9Vδ2 T cells was maintained in hypoxia, a state more representative of the tumor microenvironment *in vivo*. Indeed, Vγ9Vδ2 T cells expanded with IL-2 and IL-15 showed an increased cytotoxicity also at 1% O_2_, suggesting that these cells have the potential to be more effective in a human ACT setting as well.

To implement the use of IL-15 in clinical Vγ9Vδ2 T cell expansion protocols, cell expansion with IL-15 would first have to be tested for feasibility under conditions following the rules of good manufacturing practices (GMP). Cell expansion with IL-2 has already proven successful under GMP conditions in clinical trials using Vγ9Vδ2 T cells for ACT. In this study, we have only tested Vγ9Vδ2 T cell expansion from healthy donors. On one hand, this does not pose a problem because adoptive Vγ9Vδ2 T cell therapy potentially works in an allogenic setting, due to the independence from MHC class I presentation: A clinical study testing allogenic transfer from haploidentical donors provided very good results, as three out of four patients achieved complete remission without any signs of graft-versus-host disease (50). On the other hand, for autologous Vγ9Vδ2 T cell therapy, the feasibility of the setup would have to be confirmed with cells from cancer patients. This has previously been successful in acute myeloid leukemia patients (32) but remains to be studied for other cancer types.

In conclusion, we could show that Vγ9Vδ2 T cell expansion profited from expansion with a combination of IL-2 and IL-15 in terms of a strengthened cytotoxic response, which was also retained under hypoxic conditions. The increase in cytotoxicity went hand in hand with higher levels of T-bet as well as cytotoxic molecules perforin, granzyme B, and granulysin, indicating a contribution of these factors to the enhanced cytotoxic capacity. Further research is needed to validate if the proposed mechanism for the benefits of IL-15 signaling holds true for Vγ9Vδ2 T cells. All in all, our observations strongly suggest that future clinical protocols should include IL-15 for the *ex vivo* expansion of Vγ9Vδ2 T cells.

## Data Availability Statement

The raw data supporting the conclusions of this article will be made available by the authors, without undue reservation.

## Ethics Statement

Ethical review and approval was not required for the study on human participants in accordance with the local legislation and institutional requirements. Written informed consent for participation was not required for this study in accordance with the national legislation and the institutional requirements.

## Author Contributions

PA: study design, development of methodology, data acquisition, analysis and interpretation, and writing of the manuscript. AS and SS: data acquisition, analysis and interpretation, and revision of the manuscript. GH and PS: study supervision and design, development of methodology, data analysis and interpretation, and writing of the manuscript. All authors contributed to the article and approved the submitted version.

## Conflict of Interest

The authors declare that the research was conducted in the absence of any commercial or financial relationships that could be construed as a potential conflict of interest.

## Abbreviations

ACT: adoptive cell therapy
BrHPP: bromohydrin pyrophosphate
CAR: chimeric antigen receptor
DNAM-1: DNAX accessory molecule-1
FM-28: malignant melanoma cell line
FM-28/ZOL: FM-28 stimulated with zoledronic acid
GMP: good manufacturing practices
HMBPP: hydroxy-dimethyl-allyl-pyrophosphate
IPP: isopentenyl pyrophosphate
MFI: mean fluorescence intensity
NKG2D, natural killer group 2: member D
XBP1s: functionally spliced X-box binding protein 1
ZOL: zoledronic acid.
