# Supplementary material for: Expansion With IL-15 Increases Cytotoxicity of Vγ9Vδ2 T Cells and Is Associated With Higher Levels of Cytotoxic Molecules and T-bet
Source: Front Immunol. 2020 Aug 28;11:1868. doi: 10.3389/fimmu.2020.01868 (PMC7485111; doi:10.3389/fimmu.2020.01868)
Supplement: Supplementary file 1 [file Data_Sheet_1.PDF]

Supplementary Material

1 Supplementary Figures

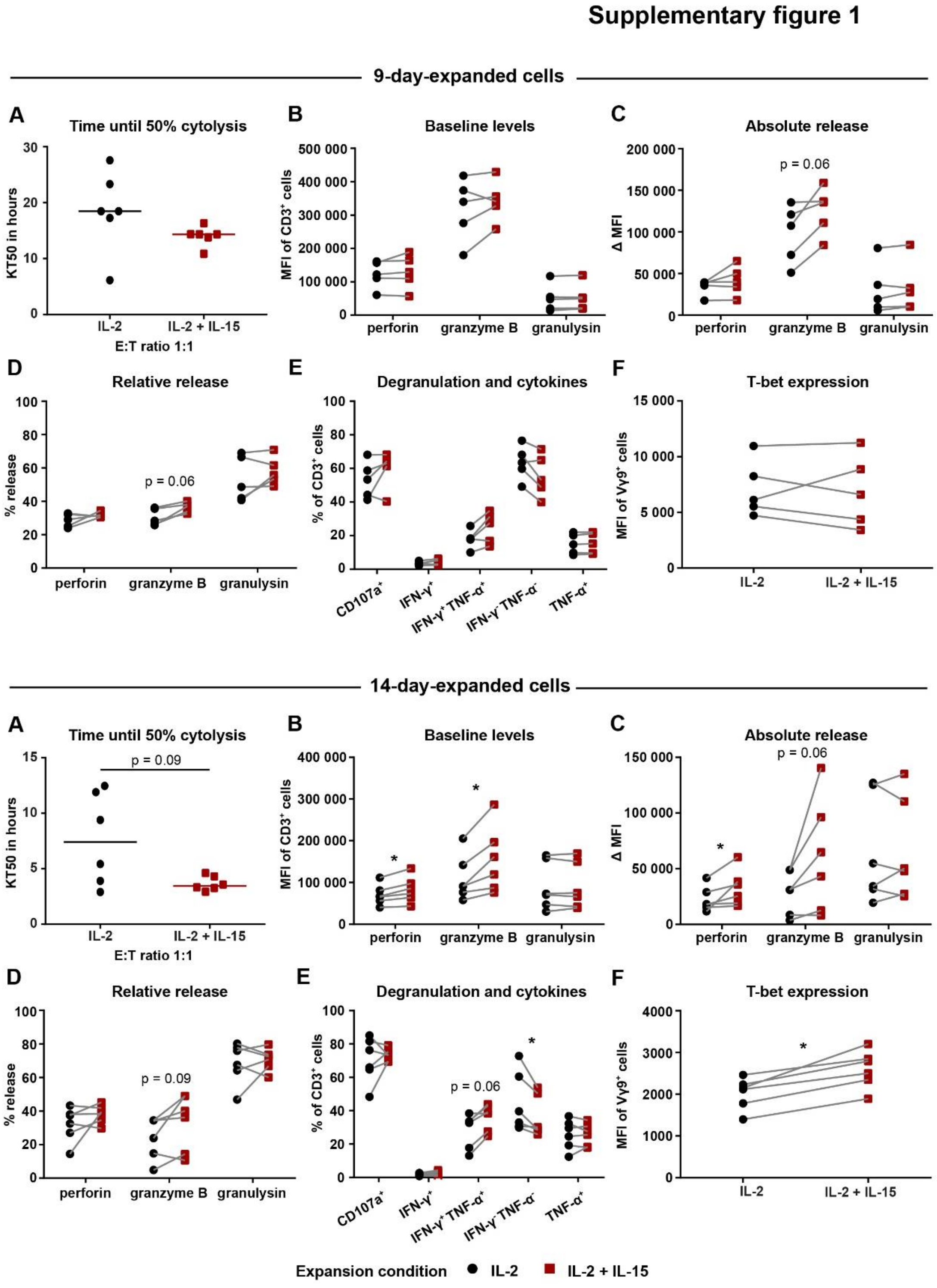

Supplementary Figure 1: Differences between IL-2-expanded and IL-2/IL-15-expanded Vγ9Vδ2 T cells are less pronounced after 9 and 14 days of culture. Vγ9Vδ2 T cells cryopreserved on day 9 or day 14 were thawed and used for an xCELLigence cytotoxicity assay against FM-28/ZOL, intracellular staining of cytotoxic molecules, degranulation markers and cytokines, as well as intracellular T-bet staining. Experiments were performed as in Figures 2, 4 and 5, respectively. Results from 9-day-expanded Vγ9Vδ2 T cells (xCELLigence: n=6 in one experiment – γδHD6,7,9-12; ICS: n=5 in one experiment – γδHD6,9-12; T-bet: n=5 in one experiment – γδHD6,7,9,11,12) are shown in the upper panel, whereas results from 14-day-expanded cells (n=6 in one experiment; γδHD6,7,9-12) are depicted in the lower panel. Black, round symbols mark IL-2-expanded cultures and red, square symbols mark IL-2/IL-15-expanded cultures. (A) Time to kill 50% of FM-28/ZOL target cells (KT50, calculated by xCELLigence RTCA Software Pro) is compared between expansion conditions. Horizontal lines indicate the median KT50. Comparison of (B) baseline levels, (C) absolute release and (D) relative release of perforin, granzyme B and granulysin between IL-2-expanded and IL-2/IL-15-expanded Vγ9Vδ2 T cells. (E) Extent of degranulation and expression of cytokines are compared between expansion conditions in the FM-28/ZOL-co-cultured Vγ9Vδ2 T cells. (F) Intracellular staining of transcription factor T-bet in IL-2-expanded versus IL-2/IL-15-expanded Vγ9Vδ2 T cells. To determine statistically significant differences between groups, the Wilcoxon matched-pairs signed-rank test was applied. Non-significant p-values greater than $p = 0.1$ are not shown. * $p < 0.05$. MFI = mean (B)/(C) or median (F) fluorescence intensity.
